# Supplementary material for: Mutation of a single cysteine in CaMKIIδ protects the heart from ischemia-reperfusion Injury
Source: bioRxiv. 2026 Apr 30:2026.04.27.721066. Preprint. [Version 1] doi: 10.64898/2026.04.27.721066 (PMC13142510; doi:10.64898/2026.04.27.721066)

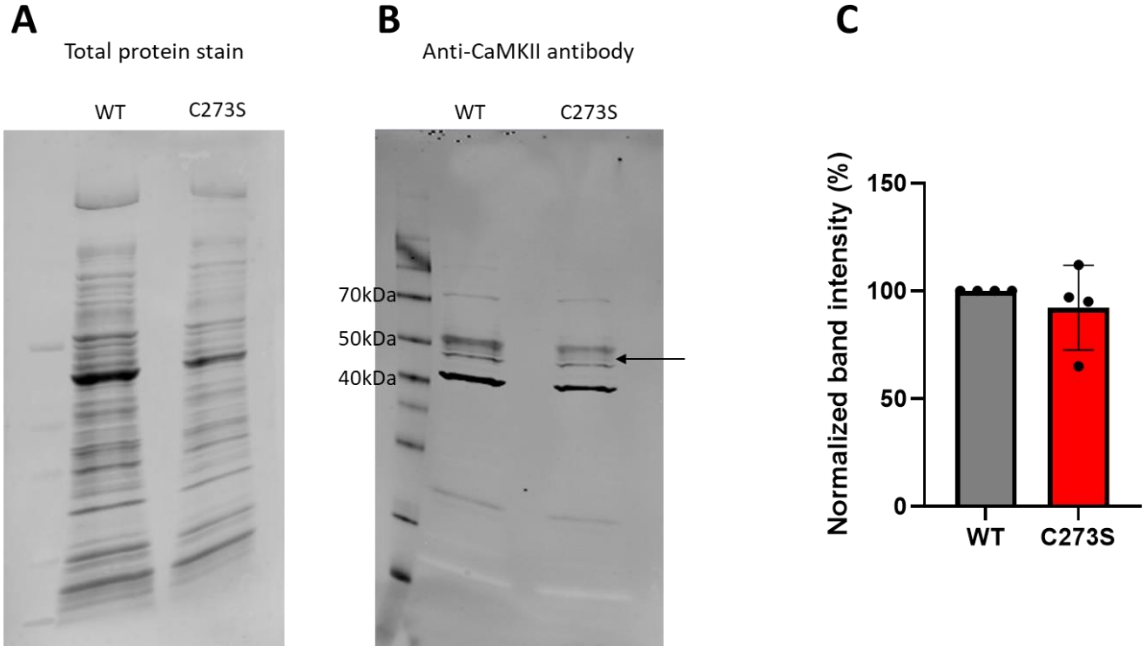


**Supplementary Figure 1. The C273S mutation did not alter CaMKII expression level in the heart. (A)** CaMKIIδC273S and wild-type mice heart lysate supernatant total protein stain. **(B)** CaMKIIδC273S and wild-type mice heart lysate supernatant were probed with anti-CaMKII antibody. Representative image of n = 4 replicates. **(C)** CaMKII band intensity around 50kDa quantification. Protein loading was normalized by total protein staining.

**Raw images**

Experiment 1

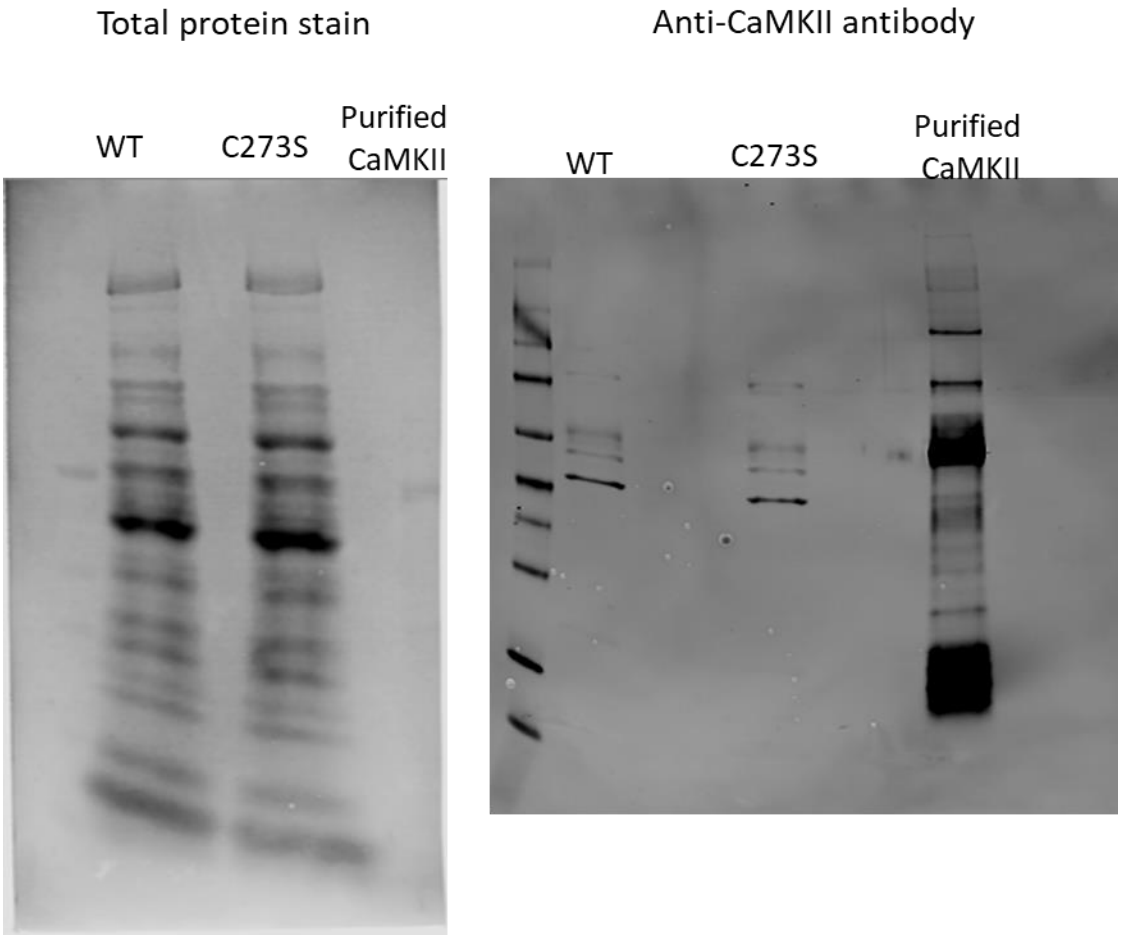

Experiment 2

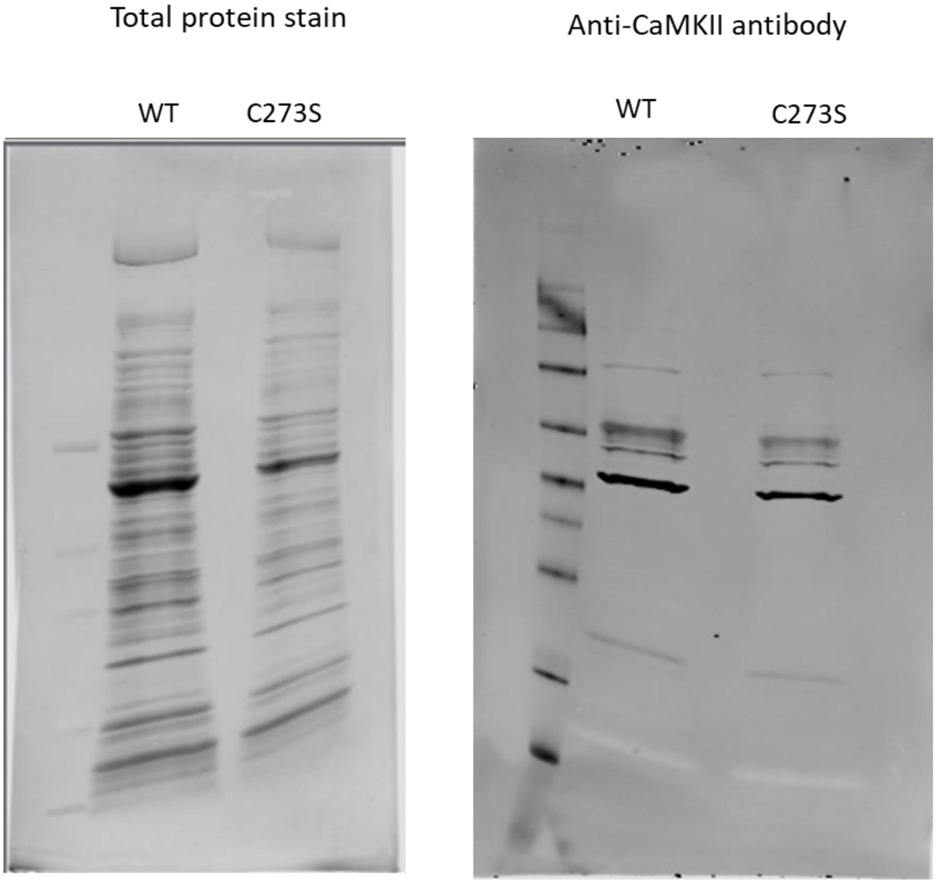

Experiment 3 and 4

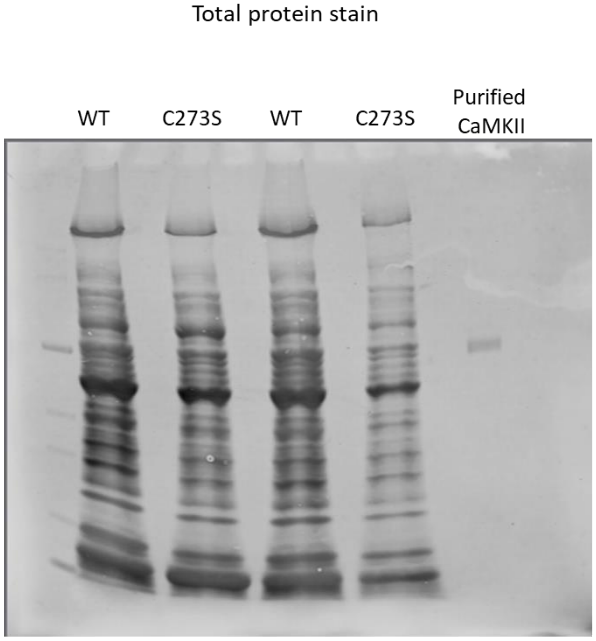


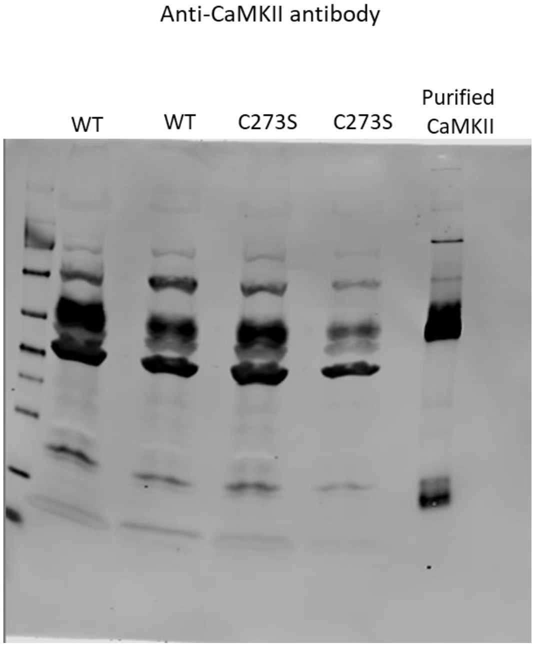

Supplement: Supplement 1 [file media-1.pdf]
